# Radiologia Brasileira: ranking among international radiology journals and among Brazilian medical journals

**DOI:** 10.1590/0100-3984.2017.50.6e1

**Published:** 2017

**Authors:** Edson Marchiori

**Affiliations:** 1 Full Professor, Universidade Federal do Rio de Janeiro, (UFRJ), Rio de Janeiro, RJ, Brazil. E-mail: edmarchiori@gmail.com

The most commonly used criterion for evaluating the quality of scientific journals is the impact factor (IF), created by the American company Thomson Reuters through the Web of Knowledge index known as Journal Citation Reports (JCR). The index is calculated by the Institute for Scientific Information (ISI) and is based on the citations, in a given year, of articles published by a given journal in the previous two years. Another metric that uses the same formula for calculating the index is the Cites per Doc (CPD) - 2 years, from the Dutch publisher Elsevier, whose indexer is Scimago, which is linked to the Scopus database. Therefore, the IF and the CPD both indicate the importance of a given journal by expressing the frequency at which the articles published therein are cited^(1)^.

In the evaluation of graduate programs in Brazil over the last four-year period, journals were classified according to their respective Scopus/Scimago journal ranking, as well as their ISI ranking, which was previously the only evaluation criterion. According to Communiqué no. 001/2012, issued by the Brazilian *Coordenação de Aperfeiçoamento de Pessoal de Nível Superior* (Capes, Office for the Advancement of Higher Education), the IF and the CPD will now both be considered for the Capes classification of journals in the area of Medicine II (which encompasses the graduate programs in radiology). For journals indexed for both databases, the highest value is considered.

The changes promoted by Capes in its system for evaluating periodicals in graduate programs brought to the national scenario in Brazil what is already a reality in the international scientific environment: the growing role of the Scopus/Scimago ranking, comparable to that of the more traditional, and until recently only, such ranking-the widely used JCR. The popularity of the Scimago database has been bolstered by the fact that it encompasses a larger number of scientific publications, including all of those indexed for ISI databases. In includes approximately twice as many journals, from three times as many countries, as does the ISI database. The Scopus (Elsevier) database is now the largest single source of peer-reviewed technical and scientific literature. Through its search mechanisms, it provides information on published articles addressing specific topics, by country, by institution, or by author. A series of studies comparing the ISI and Scimago databases did not show a clear winner, each having been found to have advantages and disadvantages. In fact, the IFs calculated by the two databases, on average, present quite similar values. It should be borne in mind that both are owned by commercial firms. However, access to the ISI database is paid, whereas the Scimago provides free access.

The measures taken by Capes have increased the importance of **Radiologia Brasileira** within the academic context in Brazil considerably, because it is indexed for the Scopus/Scimago database. In the Capes journal ranking system, known as Qualis, the journal was classified as Qualis B2 (IF between 0.8 and 1.6) for the 2012-2016 period. In the most recent Scimago evaluation^(2)^, published in September 2017, the CPD - 2 years calculated for **Radiologia Brasileira** was 2.20, putting it in 65th place among the 287 radiology journals indexed for the database. Among the 88 Brazilian medical journals indexed for Scimago, it now ranks third. It also ranks third among the 200 Scimago-indexed journals in Latin America.

We hope that these indicators of the ranking of **Radiologia Brasileira** in the national and international contexts will not be limited to mere numerical indices. Rather, they should provide the stimulus needed in order to increase the level of recognition that our journal receives within the scientific community.
